# Detection Methods for Autoantibodies in Suspected Autoimmune Encephalitis

**DOI:** 10.3389/fneur.2018.00841

**Published:** 2018-10-10

**Authors:** Gerda Ricken, Carmen Schwaiger, Desiree De Simoni, Valerie Pichler, Julia Lang, Sarah Glatter, Stefan Macher, Paulus S. Rommer, Petra Scholze, Helmut Kubista, Inga Koneczny, Romana Höftberger

**Affiliations:** ^1^Institute of Neurology, Medical University of Vienna, Vienna, Austria; ^2^Department of Neurology, Medical University of Vienna, Vienna, Austria; ^3^Department of Pathobiology of the Nervous System, Center for Brain Research, Medical University of Vienna, Vienna, Austria; ^4^Center for Physiology and Pharmacology, Medical University of Vienna, Vienna, Austria

**Keywords:** paraneoplastic neurological syndrome, autoimmune encephalitis, onconeuronal antibodies, test methods, cell-based assay, tissue-based assay, anti-neuronal antibodies

## Abstract

This review provides an overview on different antibody test methods that can be applied in cases of suspected paraneoplastic neurological syndromes (PNS) and anti-neuronal autoimmune encephalitis (AIE) in order to explain their diagnostic value, describe potential pitfalls and limitations, and discuss novel approaches aimed at discovering further autoantibodies. Onconeuronal antibodies are well-established biomarkers for PNS and may serve as specific tumor markers. The recommended procedure to detect onconeuronal antibodies is a combination of indirect immunohistochemistry on fixed rodent cerebellum and confirmation of the specificity by line assays. Simplification of this approach by only using line assays with recombinant proteins bears the risk to miss antibody-positive samples. Anti-neuronal surface antibodies are sensitive and specific biomarkers for AIE. Their identification requires the use of test methods that allow the recognition of conformation dependent epitopes. These commonly include cell-based assays and tissue based assays with unfixed rodent brain tissue. Tissue based assays can detect most of the currently known neuronal surface antibodies and thus enable broad screening of biological samples. A complementary testing on live neuronal cell cultures may confirm that the antibody recognizes a surface epitope. In patients with peripheral neuropathy, the screening may be expanded to teased nerve fibers to identify antibodies against the node of Ranvier. This method helps to identify a novel subgroup of peripheral autoimmune neuropathies, resulting in improved immunotherapy of these patients. Tissue based assays are useful to discover additional autoantibody targets that play a role in diverse autoimmune neurological syndromes. Antibody screening assays represent promising avenues of research to improve the diagnostic yield of current assays for antibody-associated autoimmune encephalitis.

## Introduction

Autoimmune diseases in the brain may affect different parts of the nervous system including neurons, glial cells or components of the blood-brain barrier. The pathobiology can be predominantly driven by T-cells or B-cells that recognize cerebral antigens. The field of autoantibody mediated autoimmune diseases of the nervous system has been expanding in the recent years, propelled by the discovery of autoantibodies against synaptic or extrasynaptic membrane antigens that lead to a new approach in diagnosing and treating patients with suspected autoimmune neurological diseases (1). While autoimmune responses against intracellular antigens are mainly associated with paraneoplastic or idiopathic neurological syndromes with poor neurological outcome, patients with surface autoimmunity show substantial response to immunotherapy (1). Cell-mediated immune attack by T-cells resulting in progressive destruction of cells is a hallmark of paraneoplastic neurological syndromes (PNS) and may explain the limited response to immunotherapy (2). Although some pathogenic impact has been described for anti-amphiphysin antibodies (3), the mechanisms and functions of other autoantibodies that evolve in the context of classical paraneoplastic syndromes (so called onconeuronal antibodies) are still poorly understood and they are rather considered as an epiphenomenon. However, the antibodies indicate the paraneoplastic etiology of the associated neurological syndrome and may serve as biomarkers for recognizing an underlying malignancy (Table 1) (4). In contrast, autoantibodies against surface antigens may directly mediate the disease (e.g., by antigenic modulation or by recruitment of immune cells or components of the complement system), among the antibodies against neuronal membrane antigens, these effects are often reversible and explain the good response to immunotherapy. Autoantibodies against cell surface antigens on neurons and glial cells can be tumor associated but derive more frequently from an idiopathic origin (1). To date, more than 16 such autoimmune syndromes are known and are summarized in Table 2. These diseases occur worldwide in diverse ethnicities and cultures. Among the anti-neuronal surface antibodies, anti-NMDAR are probably the most common ones, followed by anti-LGI1 with a reported annual incidence of 0.83 per million in one Dutch study (7). Other antibodies seem to be less frequent or their incidence has to be defined in prospective experience. Many autoimmune neurological or demyelinating syndromes are currently considered as antibody negative despite some evidence that they are antibody-mediated. Among these are patients with suspected but yet unknown antigenic targets, and further studies are required to discover these. Nevertheless, a substantial fraction of seronegative patients may harbor known autoantibodies that could be detected with a more thorough testing strategy. The following review gives an overview of the most widely used test methods and their limitations in the detection of autoantibodies and provides an outlook on possible novel approaches that are able to broaden the spectrum of identifyable antibodies.

**Table 1:** Antibodies targeting intracellular antigens.

**Intracellular antigen**	**Associated tumor**	**Main syndrome**	**Most widely used test methods**
**CLASSIC ONCONEURONAL ANTIBODY**			
Hu (ANNA1)	SCLC	Enzephalomyelitis, PCD, LE, brainstemencephalitis	Fixed TBA, LA/IB
Ri (ANNA2)	Mammary, SCLC	Brainstemencephalitis, OMS	Fixed TBA; LA/IB
Yo (PCA1)	Ovary, mammary	PCD	Fixed TBA; LA/IB
CV2 (CRMP5)	SCLC, thymoma	Encephalomyelitis, optic neuropathy, PCD, LE	Fixed TBA; LA/IB; fixed CBA
Amphiphysin	SCLC, mammary	SPS, rigidity, encephalomyelitis	Fixed/unfixed TBA; LA/IB
Ma-1/2	Testis, adenocarcinoma lung	LE, brainstemencephalitis	Fixed TBA; LA/IB
DNER/TR	Hodgkin	PCD	Fixed/unfixed TBA; LA/IB; fixed CBA
**NON-PARANEOPLASTIC ANTIBODY**			
GAD65/67	Rarley	SPS, cerebellar ataxia, LE, epilepsy	Fixed TBA; LA/IB, fixed CBA, RIA, ELISA
**TUMOR MARKERS**			
SOX1 (AGNA)	SCLC	Encephalomyelitis, PCD	Fixed TBA; LA/IB
ZIC4	SCLC	Cerebellar ataxia	Fixed TBA; LA/IB

*ANNA, anti-neuronal nuclear antibody; SCLC, small cell lung cancer; PCD, paraneoplastic cerebellar degeneration; LE, limbic encephalitis; TBA, tissue based assay; LA/IB, line assay/immunoblot; OMS, opsoclonus-myoclonus syndrome; PCA, purkinje cell autoantibody; CRMP, collapsin response mediator protein; SPS, stiff-person syndrome; DNER, delta/notch-like epidermal growth factor-related receptor; CBA, cell-based assay; GAD65, glutamic acid decarboxylase 65; RIA, radioimmuno assay; ELISA, enzyme-linked immunosorbent assay; AGNA, anti-glial nuclear antibody; ZIC4, zinc-finger protein 4*.

**Table 2:** Antibodies against surface antigens.

**Antigen**	**Tumor**	**Main clinical symptoms**	**Predominant antibody subclass**	**Most widely used test methods**
**CENTRAL NERVOUS SYSTEM**				
NMDAR	Ovarian teratoma (58% in patients >18 years)	Encephalitis	IgG1	Unfixed TBA; live/fixed CBA
LGI1	Rarely (thymoma)	LE, faciobrachial dystonic seizures, hyponatremia	IgG4/IgG1	Unfixed TBA; live/fixed CBA
CASPR2	Thymoma (38%)	LE, cerebellar ataxia, Morvan syndrome, peripheral nerve hyperexcitability	IgG4/IgG1	Unfixed TBA; live/fixed CBA
AMPAR	SCLC, breast, thymoma (60%)	LE, psychosis	IgG1	Unfixed TBA; live/fixed CBA
GABA_B_R	SCLC (50%)	LE, ataxia	IgG1	Unfixed TBA; live/fixed CBA
GABA_A_R	Thymoma, others (25%)	Status epilepticus, seizures, encephalitis	IgG1	Unfixed TBA; live CBA
mGluR1	Hematologic diseases (30–40%)	Cerebellar ataxia	NA	Unfixed TBA; live/fixed CBA
mGluR5	55% paraneoplastic (Hodgkin, SCLC)	Limbic dysfunction, movement disorders;	IgG1/IgG3	Unfixed TBA; live/fixed CBA
DPPX (Kv4.1)	Follicular B cell lymphoma, CLL	Hallucinations, agitation, myoclonus, tremor, seizures, diarrhea	IgG4/IgG1	Unfixed TBA; live/fixed CBA
IgLON5	–	Non-REM and REM-sleep disorder, brainstem and limbic dysfunction	IgG4/IgG1	Unfixed TBA; live/fixed CBA
GlyR	Lung cancer	SPS, PERM, epilepsy	IgG1	Unfixed TBA; live CBA
Dopamine 2R	–	Basal ganglia encephalitis, Sydenham's Chorea	NA	Unfixed TBA; live CBA
Neurexin3alpha	–	Seizures, orofacial dyskinesias	IgG1	Unfixed TBA; fixed CBA
PQ-type VGCC	SCLC	LEMS, PCD	NA	RIA
**ANTIBODIES IN DEMYELINATION**				
AQP4	Rarely	NMOSD, LETM, ON	IgG1	Unfixed TBA; live/fixed CBA; ELISA
MOG	–	ADEM, ON, LETM (conus), TM, NMOSD, seizures	IgG1	Live/fixed CBA
**Antigen**	**Associated diseases**	**Main syndrome**	**Predominant antibody subclass**	**Most widely used test methods**
**PERIPHERAL NERVOUS SYSTEM**				
Neurofascin155		Atypcial CIDP with distal sensomotoric neuropahty, tremor, ataxia, CNS-demyelination	IgG4	Unfixed TBA; fixed CBA; teased fibers; ELISA
Neurofascin186	IgG4-related disease; nephrotic syndrome	Subacute onset, severe phenotype, sensory ataxia	IgG4/IgG3	Fixed CBA; teased fibers; ELISA
Contactin1	Rarely nephrotic syndrome	Atypical CIDP with GBS-like onset, tremor, ataxia	IgG4/IgG3^*^	Unfixed TBA; fixed CBA; teased fibers; ELISA
CASPR1		CIDP, GBS, neuropathic pain	IgG4, IgG3^*^	Unfixed TBA; fixed CBA; teased fibers

* *IgG3 were found in patients with GBS or in the acute phase of CIDP and may switch to IgG4 in the chronic phase of the disease (5, 6). NMDAR, N-methyl-D-aspartate receptor; TBA, tissue based assay; CBA, cell-based assay; LGI1, leucine-rich glioma-inactivated 1; LE, limbic encephalitis; CASPR2, contactin-associated protein-like 2; AMPAR, amino-3-hydroxy-5-hydroxy-5-methyl-4-isoxazolepropionic acid receptor; GABA A/B R, gamma-aminobutyric acid A/B receptor; mGluR1/5, metabotropic glutamate receptor type 1/5; NA, not available; DPPX, dipeptidyl-peptidase-like protein-6; CLL, chronic lymphatic leukemia; GlyR, Glycine receptor; SPS, stiff-person syndrome; PERM, progressive encephalomyelitis with rigidity and myoclonus; P/Y-type VGCC, P/Q-type voltage gated calcium channel; LEMS, Lambert-Eaton myasthenic syndrome; RIA, radioimmuno assay; AQP4, aquaporin 4; NMOSD, neuromyelitis spectrum disorder; LETM, longitudinally transverse myelitis; ON, optic neuritis; MOG, myelin oligodendrocyte glycoprotein; ADEM, acute disseminated encephalomyelitis; TM, transvers myelitis; CIDP, chronic inflammatory demyelinating polyneuropathy; GBS, Guillain-Barré syndrome*.

## Autoantibodies in classical paraneoplastic and non-paraneoplastic neurological syndromes

### Background

**Figure 1:**
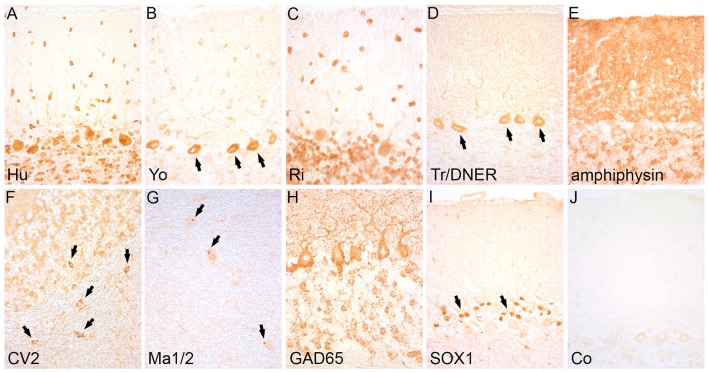
Staining pattern of antibodies targeting intracellular antigens. Indirect immunohistochemistry (avidin-biotin peroxidase method) on rat cerebellum shows a specific staining pattern of intracellular antibodies: **(A)** Anti-Hu-antibodies label the cytoplasm and nuclei of Purkinje and granule cells. **(B)** Anti-Yo antibodies show labeling of the cytoplasm of Purkinje cells (arrows) and stellate and basket cells in the molecular layer. **(C)** Anti-Ri-antibodies show the same staining pattern like Hu-antibodies in the cerebellum (differentiation is possible by staining enteric neurons of the gut that are positive with anti-Hu but negative with anti-Ri-antibodies). **(D)** Anti-Tr/DNER antibodies strongly label the Purkinj cell somata and dendrites (arrows). **(E)** Anti-amphiphysin antibodies show an intensive synaptic staining pattern in the molecular layer of the cerebellum. **(F)** Anti-CV2-antibodies mark a subgroup of oligodendrocytes in the cerebellar cortex and white matter (arrows). **(G)** Anti-Ma1/2-antibodies show a dot-like staining pattern in large neurons of the brainstem (arrows). **(H)** Anti-GAD65-antibodies display a dot-like staining of the base of Purkinje cells and a rosette-like staining pattern in the granular layer of the cerebellar cortex **(I)** Anti-SOX1-antibodies stain the nuclei of Bergmann glia in the cerebellar cortex (arrows). **(J)** Serum of a healthy control remains negative. Magnification: **(A–J)**: x400.

Since the 1980s, detailed clinical and immunological studies revealed several autoantibodies against intracellular antigens that are associated with specific paraneoplastic or idiopathic neurological syndromes (8–15). Antibodies directed against intracellular antigens usually recognize linear epitopes that can be detected by methods such as western blot analysis, line assays, enzyme-linked immunosorbent assay (ELISA), fixed tissue- (fixed TBA) or cell-based assays (CBA), or radioimmunoassay (RIA). In clinical laboratories, line assays and fixed TBAs are used most frequently. For line assays, purified recombinant proteins (e.g., paraneoplastic antigens such as Yo, Hu, Ri, CV2/CRMP5, and others) are applied on blot strips and incubated with the patient's serum or CSF. Line assays are commercially available and include most of the currently known well-characterized autoantibodies that are screened within one test run. The fixed TBAs use paraformaldehyde-fixed rodent (mouse or rat) or monkey tissue (cerebellum and enteric nervous system). The fixation is necessary for the intracellular antigen retrieval. Autoantibodies are defined as well-characterized if the serum or CSF produces a recognizable staining pattern in the fixed TBA (e.g., selective staining of Purkinje cells with Yo-positive patient's serum) (Figures 1A–J) and the antibody specificity is confirmed with the recombinant line assay (16).

### Challenges in antibody detection

#### Well-characterized onconeuronal antibodies

To provide highest sensitivity and specificity for onconeuronal antibody testing, it is recommended to combine a fixed TBA and a line assay (16). Line assays may be more sensitive in some patients than indirect immunohistochemistry (17), in addition they can help to specify the onconeuronal antibody. Using the TBA alone has the disadvantage that concomitant antibodies such as anti-nuclear antibodies may mask the immunohistochemical staining pattern. On the other hand, commercial line assays may sometimes produce reactivity in control sera without reported cancer (18) and the clinical significance is unclear. Moreover, a recent study reported that the use of commercial line assays with recombinant protein harbors the risk to miss autoantibodies as it has been shown in 4 out of 53 patients with CV2/CRMP5-antibodies (19). It was hypothesized that the epitope repertoire of the CV2 antibodies that were missed in the line assay may be different from the typical CV2 antibodies (19).

#### Tumor- and non-tumor associated intracellular antibodies

Importantly, the fixed TBA is also able to detect rare antibodies such as for example anti-protein-kinase Cgamma (PKCgamma) (20), anti-carbonic anhydrase-related protein VIII (CARP VIII) (21) or anti-rhoGTPase-activating protein 26 (ARGHAP26) (22) that bind intracellular proteins highly expressed in Purkinje cells and were originally identified in patients presenting with subacute autoimmune cerebellar ataxia. Currently, the detection of these antibodies is only possible with in-house assays and the results of the TBA can either be confirmed with in-house immunoblots or fixed cell-based assays. The PKCgamma, CARP VIII and ARGHAP26 are potentially paraneoplastic antibodies and a positive antibody-test should prompt tumor search. Recently, a novel astrocytic IgG autoantibody targeting glial fibrillary acidic protein (GFAP) has been identified in the CSF and serum of 16 patients with relapsing steroid-responsive meningoencephalitis with or without myelitis and was clinically characterized in a series of 102 patients (23, 24). The antibody was identified in the TBA showing an immunofluorescence staining of a subpopulation of astrocytes confined to pia, subpia, midbrain foci, periventricular region and rostral migratory stream and subsequently characterized in the fixed CBA as GFAP-specific. An underlying tumor can be found in 22% of patients, which include teratoma, carcinoid, salivary pleomorphic adenoma, prostate carcinoma, and melanoma. Some patients may have coexisting antibodies such as anti-NMDAR or aquaporin-4 (AQP4) antibodies, which may indicate an underlying teratoma. Although the antigen is intracellularly located, patients show good response to immunotherapy. Future investigations are necessary to clarify the role of antibodies in disease evolution, give insight into T-cell antigen specificities, and reveal possible genetic factors.

#### Anti-GAD antibodies

The glutamic acid decarboxylase (GAD) is an enzyme that catalyzes the transformation of glutamate into gamma-amino-butyric-acid (GABA). Two isoforms have been described, the 65 and the 67 kDa isoform. Both can be found in GABAergic neurons in the brain, the 65 kDa isoform is additionally expressed in islet cells of the pancreas. Low titers of GAD65 antibodies can occur in about 1% of healthy controls and in up to 80% of patients with diabetes mellitus type I (25). Currently available commercial test methods focus on the detection of the GAD65 isoform and include ELISA, radioimmunoassay, TBA, and line assays. The ELISA and RIA are more sensitive than TBA or line assays and can detect very low titers of GAD65, however, only high titers (usually >2,000 U/ml) are considered to be associated with autoimmune neurological disorders including stiff-person syndrome, ataxia, epilepsy, limbic encephalitis, and other syndromes (25). It has long been believed that screening for GAD65 antibodies is sufficient for identifying patients with GAD-autoimmunity. Interestingly, a recent study with GAD65-antibody positive patients with neurological disorders reported that GAD67 antibodies were present in the CSF even if the serum was negative for GAD67 antibodies, indicating an intrathecal antibody synthesis (26). Later it has been shown that few patients harbor antibodies only against the GAD67 isoform. The clinical picture of patients with GAD67 antibodies in serum and/or CSF is currently believed to be indistinguishable from the phenotype associated with GAD65 antibodies but the patients would be missed if GAD65 specific assays are employed such as line assays or RIA (27). Currently GAD67 can only be detected by in-house assays that either use TBA, in-house immunoblots or fixed cell-based assays.

## Autoantibodies in anti-neuronal and anti-glial surface autoimmunity

### Background

Autoantibodies directed against surface antigens often recognize conformation dependent epitopes and their detection depends on methods that preserve the three-dimensional structure of the antigen such as CBA or unfixed/postfixed TBA. In clinical laboratories, CBAs are used most frequently. The CBA consists of human or murine cells that are transfected with human complementary DNA (cDNA) and express the target antigen on their surface. Sera or CSF from patients are evaluated for the presence of antibodies by binding to these expressed antigens. CBAs are commercially available and either offered as set that allows screening of several autoantibodies within one test run [e.g., combined testing of NMDAR, AMPAR, GABA(B)R, LGI1, CASPR2, and DPPX] or as individual tests (e.g. IgLON5). The unfixed/postfixed TBAs use rodent (mouse or rat) brain tissue that contains the hippocampus and cerebellum. Sera or CSF from patients are evaluated for the presence of antibodies by binding to the rodent brain tissue and subsequently visualized either via an avidin-biotin method and light microscopy or immunofluorescence. This approach has been successful in discovering most of the autoantibodies described in the past decade. The TBAs for testing surface antibodies are commercially available or can be produced in-house and can be used as screening tool or to confirm the results of the CBA.

### Challenges in antibody detection

#### Selection of the appropriate assay

One of the first neurological autoimmune diseases that were defined by the presence of pathogenic surface autoantibodies was myastenia gravis associated with anti-acetylcholine receptor antibodies (AChR) (28). Later, surface antibodies to the P/Q type voltage-gated calcium channel (PQ-type VGCC) were identified in patients with Lambert-Eaton myasthenic syndrome (LEMS) (29, 30). Both antibodies were discovered by using RIA assays in which the antigens were labeled with ^125^I-specific neurotoxins and precipitated with patient's antibodies (31). Synthetic peptide binding studies in LEMS patients demonstrated that three epitope regions of the external linker peptides S5-S6 of domain II and IV of the alpha-1A subunit of the PQ-type VGCC were essential for creating reactivity in 9/12 patients. These epitopes are considered to be linear and test methods that lack correct membrane topology are suitable for their detection (32). In contrast, other pathogenic surface antibodies mostly recognize conformational epitopes and test methods that measure antibodies against linear or refolded epitopes often produce contradictory results, including variable frequencies of seropositivity in patients with diverse clinical syndromes and healthy controls. The RIA may give false positive results due to two issues: (1) the availability of intracellular epitopes may pick up irrelevant antibodies. For example only 56% of the serum samples that were tested positive in a RIA for voltage-gated potassium channel (VGKC) complex antibodies contained antibodies against the extracellular domain of LGI1 or CASPR2, while a considerable amount of LGI1/CASPR2-negative samples were directed against cytosolic epitopes of the VGKC (33, 34). (2) False positive results may also derive from the presence of autoantibodies against the ^125^I-neurotoxin itself (33) and false negative results may derive from an overlap of the antibody binding epitope with the binding site for the ^125^I-neurotoxin, a known phenomenon in mysthenia gravis and AChR antibodies (35–37). These difficulties emphasize the importance of test validation with different screening methods that ensure the exclusive recognition of the conformational epitope of the respective antigen and excludes interference with confounding components in the assay such as neurotoxins. The CBA allows the screening for conformation-dependent antibodies and enables the unequivocal identification of a specific surface antibody. The sensitivity of the CBA can be increased with different strategies such as 1. Using live CBAs instead of fixed cells as fixation methods may damage some epitopes (see Table 1), 2. Clustering of the antigen at high densitiy for example by co-transfecting clustering proteins such as rapsyn in the clustered AChR antibody assay (38) or 3. Increasing the number of recognizable antigens by adding further subunits of a receptor such as e.g., the gamma2 subunit of the GABA(A)R (39). The disadvantage of live CBAs is that they are technically demanding and time-consuming and their use is limited to specialized centers. The commercial CBAs are used by most clinical laboratories, however, not all antibodies can be tested with this method so far, either because the antibodies were only recently discovered and commercial CBAs may not be (readily) available, or the development of commercial assays is challenging due to methodological issues or the lack of sufficient numbers of positive controls. Another method that allows the screening for conformation-dependent antibodies is the unfixed/postfixed TBA. The TBA is a highly sensitive test method and can be used for initial screening and subsequent confirmation of positives by an antigen-specific CBA, may help to confirm the result of the CBA in case of doubtful results and is able to identify novel antibodies. A systematic comparison of the sensitivity and specificity of TBA and CBA was performed in a single-center study for anti-NMDAR antibodies and found an equal sensitivitiy for TBA and CBA (100%) in CSF, while in serum TBA was more sensitive (91.6%) than fixed CBA (86.8%) (40). Multicenter studies will be necessary to compare different assays for more target antigens and to evaluate assay reliability and reproducibility.

#### Search for antigenic targets in autoimmune neurological diseases

Some patients with autoimmune neurological syndromes remain antibody negative despite some evidence that they are antibody-mediated. Unfixed/postfixed TBAs can detect most of the currently known surface antibodies involved in autoimmune encephalitis such as NMDAR, AMPAR, LGI1, CASPR2, GABA(B)R, GABA(A)R, mGluR1, mGluR5, DPPX, Tr/DNER, Neurexin3alpha, and IgLON5 (41). In addition, anti-glial antibodies such as AQP4 antibodies can be detected (42). One limitation may be that the unfixed TBA is based on rodent brain, and antibodies that recognize only human-specific epitopes may not be detected. This is the case in most patients with anti-myelin-oligodendrocyte-glycoprotein (MOG)-antibodies (43). Furthermore, some limitations in the detection by unfixed/postfixed TBA have been described for autoantibodies against the dopamine2 receptor (D2R), glycine receptor (GlyR), and P/Q-type VGCC that are only poorly visible with this technique (41) As a consequence, it is recommended to use specific CBAs (GlyR, MOG, D2R) (44–46) or a RIA (P/Q-type VGCC) (47) for the detection of these antibodies. A potential limitation may be that some antibodies require the use of live CBAs and fixation-dependent staining protocols are inappropriate to reveal a specific antigenic epitope (see Table 2).

#### Search for antigenic targets in demyelinating diseases

Anti-AQP4- and anti-MOG-antibodies are autoantibodies against glial cells that are associated with a specific spectrum of demyelinating diseases. Anti-AQP4-antibodies were the first antibodies with a clearly defined target that were identified in patients with demyelinating diseases (48) and now serve as biomarker for the diagnosis of patients with neuromyelitis optica spectrum disorders (NMOSD) (49). The incidence of AQP4-antibody positive NMO ranges from 0.05 to 0.4 per 100,000 (50). The antibodies were originally discovered by using indirect immunofluorescence on rodent brain tissue showing a characteristic staining pattern of astrocyte end feet around blood vessels, along the pial surfaces and Virchow-Robin spaces (48, 51). Meanwhile, the standard for most clinical laboratories for testing AQP4-antibodies is the use of CBAs either in form of commercially available fixed CBAs with the AQP4-M1 isoform or in-house live CBAs using the AQP4-M23 isoform. A large multicenter study systematically compared different AQP4 assays including CBAs, TBAs, flow cytometry, and ELISA and found the CBA as most sensitive and specific test method, with some benefit of using the AQP4-M23 isoform and additionally described high sensitivity and specificity for immunohistochemistry and flow cytometry in some specialized centers (52). Recently, the search for novel antibodies in demyelinating diseases by using monoclonal recombinant antibodies from patients with NMOSD revealed an anti-endothelial cell antibody against the endoplasmic reticulum chaperon GRP78 that may compromise the blood-brain barrier (53). Further studies will be necessary to clarify a potential role in initiating the inflammatory cascade and disease activity of NMOSD.

Anti-MOG antibodies were defined in patients with acute disseminated encephalomyelitis (ADEM), uni- or bilateral optic neuritis, transverse myelitis, longitudinally extensive transverse myelitis, and neuromyelitis optica. In children, one third of patients with an acute demyelinating syndrome are MOG-antibody positive (44, 54, 55). Most of the patient's antibodies recognize a human-specific epitope and TBAs based on rodent tissue are not suitable for their detection. Human MOG-antibodies were recently tested on human brain tissue and 88% of samples showed a staining of white matter (56), this approach could provide a promising screening tool in the search for novel antibodies. Currently anti-MOG-antibodies are either tested with commercial or in-house live CBAs employing HEK cells transfected with full-length human MOG (57–59). Further multicenter studies of different assays will be necessary to compare the sensitivity and specificity and identify difficulties in different test methods.

#### Search for antigenic targets in paranodopathies—a novel subgroup of autoimmune peripheral neuropathies

**Figure 2:**
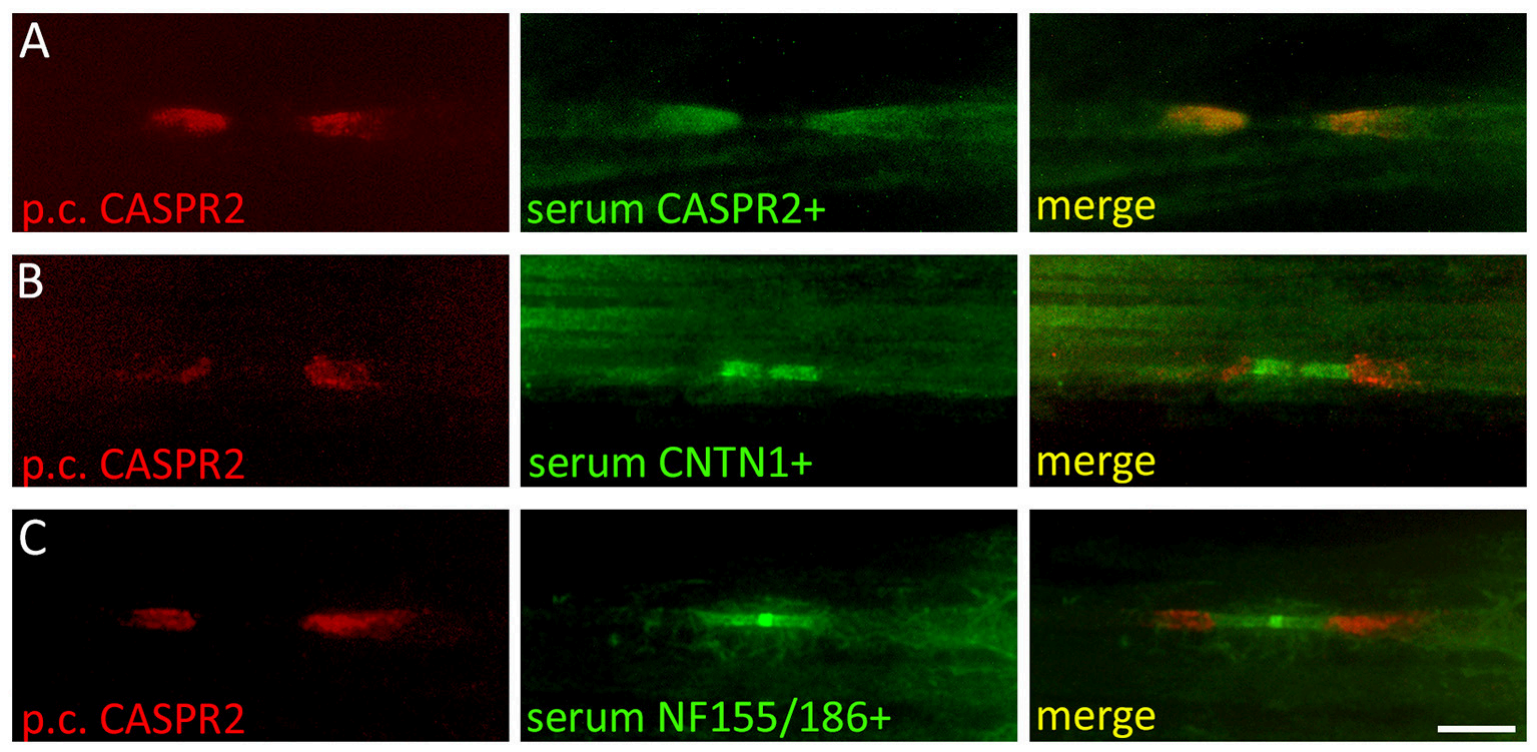
Screening of autoantibodies on teased nerve fibers in patients with peripheral neuropathies. Rat sciatic nerve fibers were immunostained with a polyclonal rabbit anti-CASPR2 antibody (red) and serum from a patient with **(A)** anti-CASPR2 antibodies (green), **(B)** anti-contactin1 antibodies (green), and **(C)** anti-neurofascin155/186 antibodies (green). CASPR2 labels the juxtaparanodal region of the node of Ranvier, contactin1 the paranodal and neurofascin155/186 the paranodal and nodal region. CNTN1, contactin1; NF155/186, neurofascin155/186; Scale bar = 10 μm.

In patients with autoimmunity that primarily affects nervous tissue outside the CNS, the TBA can be expanded to the respective target region. For example, in patients with peripheral neuropathy, screening on teased sciatic nerve fiber preparations from rodents can detect antibodies against proteins in the node of Ranvier (Figures 2A–C) (60, 61, 5). The node of Ranvier is a highly specialized structure that is important for the saltatory conduction of impulses in myelinated nerve fibers. A large number of adhesion molecules are involved in the formation of the axon-myelin junctions and compartmentalization of voltage-gated potassium channels and serve as potential target for autoimmunity (62). Autoimmune diseases associated with antibodies against proteins in the paranodal region of the node of Ranvier are subsumed as paranodopathies and define an exciting group of autoimmune peripheral neuropathies clinically presenting as atypical chronic inflammatory demyelinating polyneuropathy (CIDP) or Guillain-Barré-syndrome (GBS) that may benefit from treatment with rituximab (Table 2) (63). Based on results of teased nerve fiber screening, it is supposed that up to 40% of CIDP patients harbor antibodies against components of the myelin or the axon (64). Some antibodies such as anti-neurofascin155 or anti-contactin1 are detectable in teased nerve fibers and in the unfixed TBA (hippocampus and molecular layer of cerebellum) (61, 65, 6), while others may only be detectable in teased nerve fibers (5).

#### Significance of primary cell cultures in suspected autoimmune encephalitis

**Figure 3:**
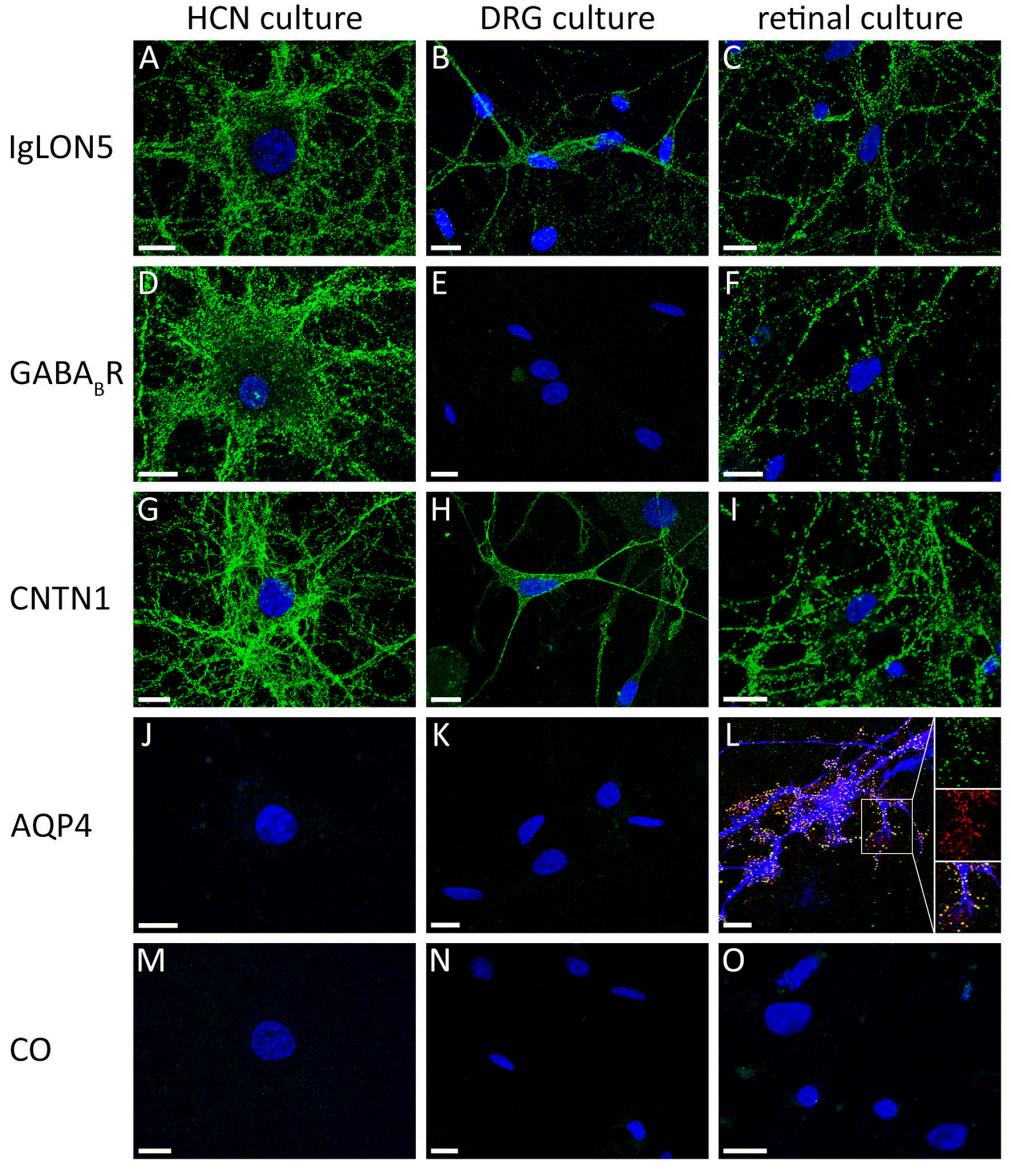
Comparison of reactivity of different antibodies against cell surface antigens on different primary neuronal and glioneuronal cell cultures. **(A)** The serum of a patient with anti-IgLON5 antibodies shows an intensive labeling of live nonpermeabilized rat hippocampal neurons, **(B)** rat dorsal root ganglion cells (DRGs), and **(C)** dissociated rat retinal cell culture. In contrast, **(D)** the serum of a patient with anti-GABA(B)R antibodies labels hippocampal neurons but not **(E)** DRGs. **(F)** The retinal cell culture is strongly GABA(B)R positive. **(G–I)** A serum of a patient with anti-contactin1 antibodies labels all three types of cell cultures. A serum of a patient with **(J)** anti-aquaporin4 antibodies is negative on hippocampal neurons and **(K)** DRGs, but **(L)** labels the end feet membranes of GFAP-positive Müller cells (red: rabbit polyclonal anti-AQP4 antibody; green: serum of a patient with AQP4 antibodies; blue: mouse monoclonal anti-GFAP antibody). **(M–O)** A healthy control is negative. HCN, hippocampal neurons; DRG, dorsal root ganglion cells; retinal culture, dissociated rat retinal cell culture; CNTN1, contactin1; AQP4, aquaporin-4; CO, healthy control; Scale bar = 10 μm.

A complementary method to the screening of surface antibodies on tissue based assays are live cultures of neurons. These neurons can be used to identify a binding between an individual's antibody and a specific surface peptide on the intact neuronal membrane. A positive staining of the cells gives evidence that the detected autoantibody recognizes a surface antigen and is likely to play a pathogenic role in the disease (41). This method is used in research laboratories and may (1) help in the diagnostic procedure to differentiate between surface or intracellular reactivity in samples with doubtful results in the TBA and (2) can be used to identify the target antigen by performing immunoprecipitation of the patient's serum together with the cell culture and subsequently identify the co-precipitated target antigen by mass spectrometry. Rat hippocampal neurons are the most frequently used cell culture system for the visualization of anti-neuronal surface antibodies, however, not all neuronal surface proteins are expressed in these cells and the absence of binding should not necessarily imply the absence of surface reactivities. Moreover, anti-glial antibodies are not displayed. Alternatively, other neuronal or mixed glioneuronal cell cultures may be useful to demonstrate a neuronal or glial surface autoantibody. Anti-contactin1 and anti-CASPR1 antibodies were shown to label both rat hippocampal neurons and dorsal root ganglion cells (5, 66), in contrast, anti-AQP4-antibodies may only be detectable in glioneuronal cell cultures including rat retinal cell cultures that contain Mueller cells (Figures 3A–O) (67, 68). The screening of samples with suspected seronegative autoimmune encephalitis on different live cell cultures might enable to broaden the spectrum of identifyable antibodies and provide a promising approach for discovering novel autoantibodies against surface antigens.

## Selection of appropriate sample types

A critical step to successfully detect anti-neuronal or anti-glial antibodies is the combined testing of serum and CSF in an individual patient. This has several reasons. First, the detectability of specific antibodies may differ between serum and CSF. Some antibodies may be easier identifyable in CSF than serum, for example antibodies against the NMDAR, GABA(B)R or AMPAR. In a study of 577 patients with anti-NMDAR encephalitis, in one out of 7 patients antibodies were only detectable in CSF and testing restricted to serum would have misdiagnosed the patients as seronegative (69). Other autoimmunities may present with a substantial systemic autoantibody production such as patients with GAD65 antibodies, but they may additionally harbor antibodies against the GAD67 isoform in CSF and few cases were described with a restricted autoimmunity to GAD67. It will be important to collect more cases with exclusive GAD67 reactivity to see whether they present specific neurological features. Finally, some antibodies are more prevalent in serum than in CSF. These are for example anti-AQP4 or anti-MOG-antibodies (44). ADEM may be an important differential diagnosis for anti-neuronal autoimmune encephalitis and the testing for anti-MOG-antibodies only in CSF may lead to false negative results and delay in diagnosis.

Second, serum and CSF might harbor different sets of antibodies and in this constellation the antibodies in CSF may correlate better with the neurological symptoms than those in serum, as it has been shown in a study of patients with GABA(A)R antibodies (70).

Third, testing of serum and CSF may have methodological implications. It has been shown that testing of antibodies only in serum harbors the risk for increased background and unspecific cross-reactivity that may result in contradictory test interpretations (71, 72). To avoid misinterpretations or delay in diagnosis the testing of both serum and CSF is recommended (73).

## Testing of the specific immunoglobulin isotypes

Antibodies in human plasma belong to different isotypes according to their type of heavy chains and include IgG, IgA, IgM, IgE, and IgD. The IgG is the most abundant antibody isotype and can be classified into four subclasses IgG1, 2, 3, and 4. Pathogenic mechanisms in anti-neuronal autoimmune encephalitis were mainly associated with antibodies of the IgG isotype that can have different effects on the targeted antigen. The IgG1-3 subclasses may alter the synaptic structure by cross-linking and internalization of the receptor such as in anti-NMDAR (74) or anti-AMPAR encephalitis (75), serve as antagonist of baclofen in anti-GABA(B)R autoimmunity (41), or reduce the amount of receptor at the synapse such as in anti-GABA(A)R autoimmunity (70). In contrast, antibodies of the IgG4 subclass mainly seem to mechanically interfere between the receptor-ligand interaction resulting in the blockade of protein-protein interaction (76). Recently, antibodies of the IgA and IgM isotype against the NMDAR were found in up to 22% of patients with different neurological diseases and in healthy controls by using fixed CBAs and it was hypothesized that the symptomatic relevance of the antibodies is related to a compromised blood-brain barrier that allows access to the brain (77–81). Moreover, it was demonstrated that NMDAR antibodies regardless of the clinical presentation of the donor (healthy or ill) and immunoglobulin class could provoke receptor internalization in human-induced pluripotent stem cell-derived neurons and reduced the glutamate-evoked currents in NMDAR expressing Xenopus oocytes (79). However, the functional significance of IgA and IgM NMDAR antibodies and their ability to internalize the NMDAR could not be confirmed in a subsequent study using CBAs, unfixed TBAs, and immunostaining of live primary hippocampal neurons (82). Since robust association with anti-NMDAR encephalitis was only shown for IgG antibodies, the antibody testing in clinical practice should be focused on the IgG antibodies.

## Summary

The expanding field of antibody-mediated autoimmunity allows the identification of a vast range of neuronal and glial autoantibodies, which enables a more precise diagnosis of specific syndromes and disease subtypes. It is important to know that testing for onconeuronal antibodies requires other methods (line assays, fixed TBAs) than surface antibodies (CBAs and unfixed/postfixed TBAs). The highest sensitivity and specificity of a test result can be achieved by cross-validation with different test methods and the combined testing of serum and CSF samples. Test results should always be interpreted in context with the clinical presentation. In case of an unexpected positive or negative result, re-testing of the sample or performing confirmatory tests might be considered. The screening for surface antibodies on unfixed TBA can detect a large number of anti-neuronal and some anti-glial antibodies with some limitation for anti-GlyR, anti-D2R, and anti-MOG-antibodies. In patients with peripheral neuropathies, the screening can be expanded to teased nerve fibers to detect antibodies against proteins of the node of Ranvier. Moreover, the staining of primary cultures of neurons or glioneuronal cell cultures may give evidence that the detected autoantibody recognizes a surface antigen and enables the characterization of novel surface antibodies. The accurate and rapid detection of autoantibodies in CSF and serum may initiate immunotherapies to improve patients outcome.

## Author contributions

GR and RH have access to all the data and take responsibility for the data, accuracy of the data analysis, design and conceptualization of the review. CS, DD, VP, JL, SG, SM, PR, PS, HK, and IK: analysis and interpretation of the data, drafting, and revising the manuscript for intellectual content.

### Conflict of interest statement

RH received speaker honoraria from Euroimmun. The Medical University of Vienna receives payments for antibody assays and antibody validation assays (aquaporin-4 and other anti-neuronal and anti-glial antibodies) organized by Euroimmun (Lübeck, Germany). The remaining authors declare that the research was conducted in the absence of any commercial or financial relationships that could be construed as a potential conflict of interest.

## Abbreviations

AMPAR: amino-3-hydroxy-5-hydroxy-5-methyl-4-isoxazolepropionic acid receptor
CASPR1/2: contactin-associated protein-like 1/2
CNTN1: contactin1
D2R: dopamine-2 receptor
DPPX: dipeptidyl-peptidase-like protein-6
GABA A/B R: gamma-aminobutyric acid A/B receptor
GAD: glutamic acid decarboxylase
GlyR: Glycine receptor
LGI1: leucine-rich glioma-inactivated 1
mGluR1/5: metabotropic glutamate receptor type 1/5
NF155: neurofascin155
NMDAR: N-methyl-D-aspartate receptor
P/Q-type VGCC: P/Q-type voltage-gated calcium channel
VGKC: voltage-gated potassium channel.
